# Study design of the InTakeCare trial: a digital health solution to monitor and improve medication adherence in hypertensive patients

**DOI:** 10.1093/ehjdh/ztag051

**Published:** 2026-03-28

**Authors:** Emanuele Tauro, Alessandra Gorini, Martina Vigorè, Lucia Zanotti, Alessandro Croce, Martino F Pengo, Davide Soranna, Antonella Zambon, Gianfranco Parati, Enrico Gianluca Caiani, Grzegorz Bilo

**Affiliations:** Department of Electronics, Information and Bioengineering, Politecnico di Milano, Milan, Italy; Department of Cardiology, Istituto Auxologico Italiano IRCCS, Milan, Italy; Dipartimento di Scienze Cliniche e di Comunità, Dipartimento di Eccellenza 2023-2027, Università Degli Studi di Milan, Milan, Italy; PsyCaRe Lab, Istituti Clinici Scientifici Maugeri IRCCS, Milano, Camaldoli, Italy; PsyCaRe Lab, Istituti Clinici Scientifici Maugeri IRCCS, Milano, Camaldoli, Italy; Department of Cardiology, Istituto Auxologico Italiano IRCCS, Milan, Italy; Department of Cardiology, Istituto Auxologico Italiano IRCCS, Milan, Italy; Department of Cardiology, Istituto Auxologico Italiano IRCCS, Milan, Italy; Department of Medicine and Surgery, University of Milano-Bicocca, Piazzale Brescia, 20, Milano 20149, Italy; Department of Cardiology, Istituto Auxologico Italiano IRCCS, Milan, Italy; Department of Cardiology, Istituto Auxologico Italiano IRCCS, Milan, Italy; Department of Cardiology, Istituto Auxologico Italiano IRCCS, Milan, Italy; Department of Medicine and Surgery, University of Milano-Bicocca, Piazzale Brescia, 20, Milano 20149, Italy; Department of Electronics, Information and Bioengineering, Politecnico di Milano, Milan, Italy; Department of Cardiology, Istituto Auxologico Italiano IRCCS, Milan, Italy; Department of Cardiology, Istituto Auxologico Italiano IRCCS, Milan, Italy; Department of Medicine and Surgery, University of Milano-Bicocca, Piazzale Brescia, 20, Milano 20149, Italy

**Keywords:** Digital health, Randomized control trial, Medication adherence, Hypertension, Personas

## Abstract

**Aims:**

The InTakeCare Trial aims to develop, implement, and clinically validate a novel digital health solution (DHS) to improve medication adherence (MA) in patients with arterial hypertension. This initiative responds to the global issue of poor MA to long-term pharmacological treatments, compromising patient outcomes and increasing healthcare costs.

**Methods and results:**

This study is structured as a multidisciplinary, single-centre, randomized, open, blinded-endpoint clinical trial. In the preliminary phase, focus groups and a survey are conducted to develop patient personas and fictional archetypes that represent real people in the development of interventions, informing the personalization of a mobile application for patients and a web dashboard for physicians. The developed DHS is designed to deliver tailored reminders, health messages, and real-time monitoring of MA. After a pilot usability phase, 206 hypertensive patients will be randomized to standard care or active intervention for a 3-month period, followed by a 2-month observational phase to assess the persistence of intervention effects on adherence. MA is measured via direct (e.g. plasma drug levels) and indirect (e.g. self-reports) methods, alongside ambulatory blood pressure monitoring and behavioural engagement scales.

**Conclusion:**

This study offers a novel, theory-informed framework for the design of personalized DHS in chronic disease management. Leveraging user-centred design and machine learning-driven persona classification, InTakeCare introduces a scalable, evidence-based solution that addresses individual variability in adherence behaviours. The trial’s findings are expected to generate insights into the efficacy, usability, and acceptability of personalized DHS in routine clinical practice, providing a replicable model for future DHS in other chronic conditions.

## Graphical Abstract

**Graphical Abstract:**
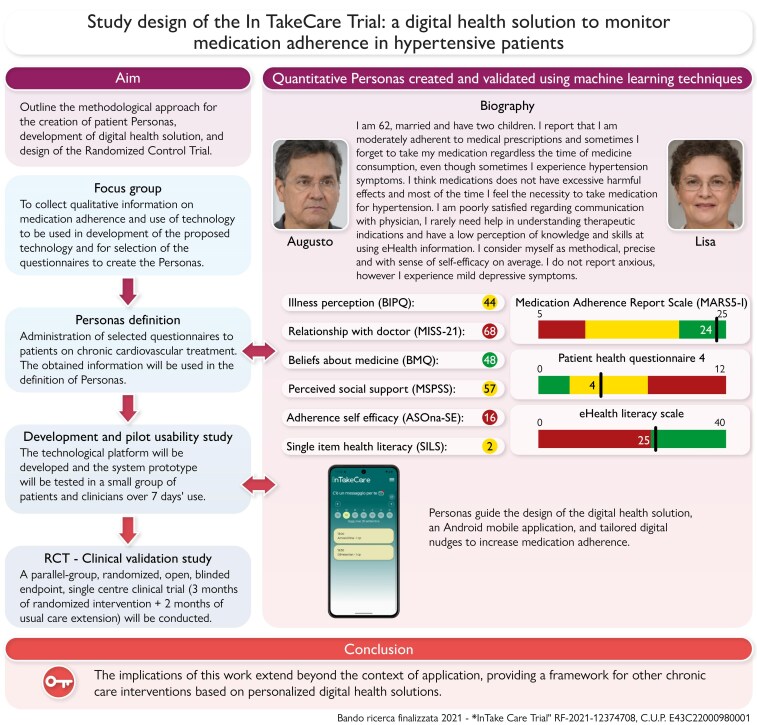

## Introduction

Inadequate medication adherence (MA) in chronic patients, including those on treatment for hypertension, is a major challenge, worsening health outcomes and costs.^1–3^ Within hypertension, MA constitutes one of the most critical aspects of disease management, as poor adherence reduces quality of life and increases hospitalization and death.^3–5^ After 1 year of treatment, MA falls to ∼50%,^6^ underscoring the need for solutions that better support therapy management. Existing approaches are largely one-size-fits-all, and insufficiently tailored to individual needs.^7,8^

The InTakeCare trial is a collaborative project between Politecnico di Milano, Istituto Auxologico Italiano, IRCCS and Istituti Clinici Scientifici Maugeri, IRCCS, involving medical, psychological, and engineering activities. The study was launched in March 2023, over 3 years, and aimed to develop and validate a digital technology to monitor and improve with a tailored approach MA in hypertension. The clinical trial was approved by the ethical committees of Istituto Auxologico Italiano (approval number 09M201, 18 April 2023) and Politecnico di Milano (approval number 23/2023, 29 June 2023) and registered on ClinicalTrials.gov (registration number NCT06229171, 29 January 2024).

The study hypothesizes that a customizable digital health solution (DHS)—a low-cost, user-friendly smartphone app plus a clinician web interface—will be useful in monitoring and supporting adherence. The intervention personalizes adherence support via (i) analysis of the sociodemographic, cognitive, and psychological characteristics of the target patient relevant to MA and (ii) allocation to predefined personas. Personas cluster hypertensive patients with multicomponent traits, enabling personalization while avoiding a one-to-one approach^9–13^; engagement content and frequency are persona specific. The project will clinically validate a DHS tailored to improve MA in hypertension.

The aim of this paper is to (i) outline the methodological approach to create patient personas and develop the DHS and (ii) present the randomized clinical trial (RCT) protocol designed to evaluate usability and efficacy of the DHS.

## Methods

**Figure 1.**
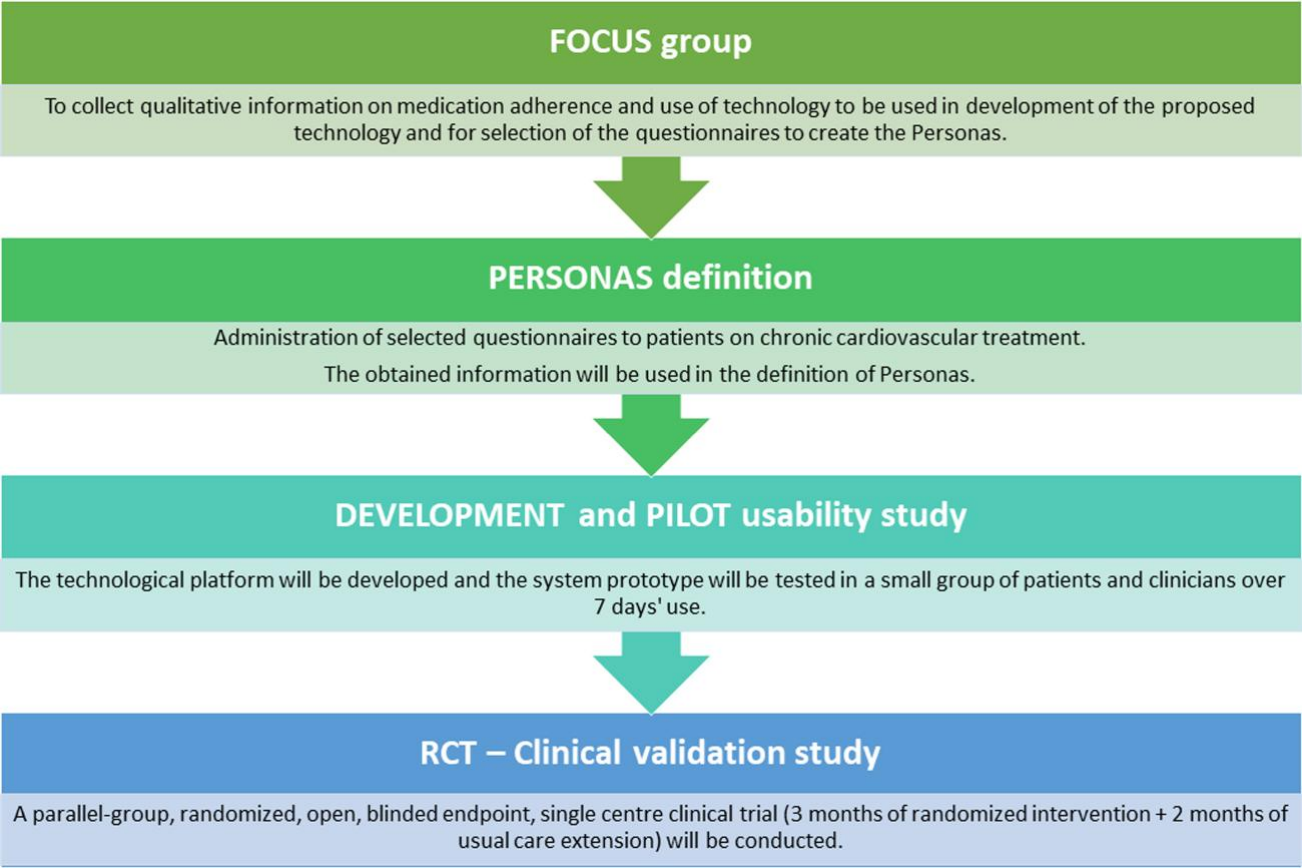
Project concept diagram, highlighting the four main steps of the InTakeCare Study and the order in which they will be performed.

The InTakeCare Trial comprises four main steps, carried out both concurrently and in sequence. First, we develop hypertensive patient personas and the InTakeCare DHS, then design the RCT protocol for clinical validation. The output of each stage informs the next. *Figure 1* shows the study flow: persona definition, a pilot usability study of the DHS, and the RCT for clinical validation.

### Study objectives

The InTakeCare trial has one primary objective and two independent secondary aims. The primary objective is to test the usability and efficacy of the developed DHS via a pilot usability study followed by an RCT in patients with arterial hypertension. Efficacy will be evaluated using indirect and direct measures of MA (including self-reported adherence, pill counts, plasma/urine drug levels and changes in 24-h ambulatory blood pressure).

The two secondary aims are to (i) create personas of hypertensive patients to tailor interactions via user-centred design and enhance sustained engagement with the developed DHS and (ii) develop a DHS comprising a web dashboard for clinicians to manage prescriptions, monitor adherence, and send personalized prompts and a patient mobile application to receive reminders, tailored messages, and communicate asynchronously with the physicians.

### Focus groups

Three focus groups conducted in the initial phase involve (i) 10 patients recruited in a high-density (HD) urban context in Milan, Italy, (ii) 10 patients recruited in a low-density (LD) urban context in Meda, Italy, and (iii) 6 physicians. The aim of the focus groups is to identify the main barriers to MA perceived by chronic patients and physicians. These barriers are structured into macro-categories according to current literature^14^: factors related to the person, factors related to the medication, and factors related to the physician–interaction. Focus groups are conducted by the psychologists involved in the study, using the Wooclap tool to create a qualitative graphical mapping of the topics identified by participants. These topics are then used to create an interactive discussion between participants and psychologists. The themes that emerge are then reported in a written report by the psychologists involved.

Focus groups also serve as a qualitative method to explore the patients’ perspectives on using technology to enhance health-related behaviours, with a specific focus on adherence, their expectations for the solution’s functionalities, as well as the physicians’ needs and expectations regarding the proposed technology. Insights gathered from these discussions inform both the development of the technology and the selection of questionnaires used to construct patient personas.

### Personas for hypertensive patients

Starting from scientific literature, a set of validated questionnaires was identified as the basis for the creation of the web survey. This web survey is further extended with a set of ad-hoc questions, based on the factors identified by the focus groups.

The aim of the web survey is to collect real-patient data to be used in the subsequent development of data-driven personas that capture a broad spectrum of behavioural, psychological, and sociodemographic characteristics relevant to MA. This approach supports the development of digital nudges and interventions aimed at personalizing the DHS and enables the inclusion of a heterogeneity of patient profiles that would not be obtainable from a small formative patient sample. Consequently, it allows to tailor the interventions without recurring to a fully individualized approach, unrealistic in clinical practice. Furthermore, the personas do not replace direct patient involvement, providing instead a structured representation of adherence-related patterns that are derived from the patients and physicians involved in the focus groups.

The web survey is divided into four blocks of questions, each focusing on a different type of information, as presented in *Table 1*. The web survey is disseminated using the survey modality of the REDCap platform^14^ both through online campaign using common communication channels, and directly to individual patients presented by the participating physicians. The survey is completely anonymous, and no identifiable personal data, including the internet protocol, are recorded or collected.

**Table 1.** Description of the blocks of questions in the web survey for medication adherence in chronic patients suffering from hypertension, highlighting the focus of collected information and the corresponding validated questionnaires.

Block of questions	Focus and validated questionnaires
**Socio-demographic characteristics and eHealth literacy**	Socio-demographic information and one validated eHealth literacy questionnaire: Socio-demographic ad-hoc eHEAlth Literacy Scale (eHEALS)^15^
**Physical health**	Questions about the physical health of the subject and related behaviours: 3 questions.
**Perception and usage of medication**	Usage of medication, adherence, strategies related to taking medication, beliefs about medicine and health literacy: Beliefs about Medicine Questionnaire (BMQ)^16^ Medication Adherence Report Scale (MARS5-I)^17^ Single-Item Literacy Screener (SILS)^18^
**Psychological and emotional factors**	Questions about the relationship with physicians and psychological and emotional factors related to medication adherence. Antecedent and Self-efficacy on Adherence (ASoNA-SE)^19^: 6 questions. Medical Interview Satisfaction Scale (MISS-21)^20^ Multidisciplinary Scale of Perceived Social Support (MSPSS)^21^. Brief Illness Perception Questionnaire (BIPQ)^22^. Patient Health Questionnaire-4 (PHQ-4)^23^

Personas are created and validated using data-driven methods already available in scientific literature,^9,24^ leveraging domain knowledge available from previous studies that addressed populations of chronically ill patients.^10,11,25^ Collected data are split into two datasets: one containing 80% of the respondents, used for persona creation, and the other containing the remaining 20%, used for persona validation. Respondents in the Personas creation dataset are preprocessed and clustered into different Personas, each highlighting different characteristics and needs related to the aims identified by the focus groups.^9^ Validation of the personas is performed using a self-supervised machine learning (SSML)-based framework developed in a previous study.^24^ This framework leverages a completely data-driven methodology that allows automatic identification of validation set patients, evaluating whether the performed clustering is capable of correctly discerning between the identified personas based on standard performance metrics such as precision, recall, and F1 score. The validation method also provides a trained model that can be used to classify new subjects into their corresponding personas, leveraging the non-linear patterns identified by the SSLM algorithm when compared to the clustering model.^24^ Given the complexity of MA behaviours, additional evaluation is required to ensure that the personas developed in this study meaningfully reflect established adherence constructs. This step is essential to ensure that the personas not only support personalization of the DHS but also maintain coherence with recognized patterns of adherence behaviour.^26–28^

Personas support the development of the platform by informing the identification of digital nudges tailored towards specific users. Digital nudges refer to strategic design elements that guide individuals towards specific behaviours or decisions without removing their possibility of choice.^29^ Within InTakeCare Trial, digital nudges are implemented through personalized messages that are sent to the user. These messages are co-designed in collaboration with psychologists and physicians, and aim at increasing the self-efficacy, health literacy, and knowledge of the pathology of chronic patients. The personalization is performed through the use of personas, that support the creation of messages targeting specifically the characteristics of the group of users they are referring to, aiming to increase their MA and improve their quality of life and expected outcomes.^30^ The frequency of the messages is defined by physicians and psychologists depending on qualitative evaluation of the Personas.

### InTakeCare platform

InTakeCare platform is a modular and scalable DHS that integrates both active and passive functionalities to ensure that patients receive essential support in adhering to their treatment plans. Active features, including reminders and therapy management tools, serve as motivational aids, facilitating patients in sustaining their progress. Passive functionalities, such as data monitoring and analysis capabilities, empower healthcare providers to promptly detect instances of non-adherence and intervene effectively.

The platform consists of an internal system and a range of external modules that work in collaboration to provide its full functionality and is designed using the three-tier architecture, a well-known and functional framework for software development.^31^ This architecture is composed of a data tier, an application tier, and a presentation tier, thus enabling easier maintenance, scalability, and management of the software. The three-tier architecture facilitates the handling of intricate workflows and interactions, contributing to the robustness of the system.^32^ Furthermore, it is based on the principles of separation of concerns^33^ and single responsibility,^34^ involving the separation of a program into distinct modules, each addressing a separate concern and being responsible exclusively for that concern.

The data tier is responsible for storing, updating, and managing the data necessary for InTakeCare to offer its functionalities. It is based on a cloud server established using a NoSQL database management system. This approach is chosen due to the inherent capabilities of non-relational databases, not necessitating strict constraints and schemas, thus enabling greater flexibility in data structure, augmented scalability of the developed system, and improved efficiency in executing queries.^35^

The application tier acts as the intermediary between the presentation and the data tiers, handling the logic, processing requests, and facilitating communication with the underlying database. InTakeCare is composed by a single scalable server that handles the full workload of the whole system, developed using the representational state transfer architecture and the NestJS framework.^36^ It is defined by a series of modular, independent components, including providers and controllers, that receive input from the presentation tier applications, perform logical operations, and facilitate communications with the underlying data tier.

The presentation tier handles the interaction between the user and the InTakeCare platform. It represents all the external modules that can interact with the internal system composed by the other two tiers, and it is thus characterized by a high range of diverse channels. For the purpose of this study, a web dashboard and a mobile application are developed. These modules allow InTakeCare to deliver a comprehensive and integrated solution that could be tailored towards different users’ preferences, encompassing patients with different lifestyles. InTakeCare allows physicians to simultaneously monitor the adherence of a large number of chronic patients without requiring excessive time or attention, leveraging its features to further tailor the content and modality of interaction for each of them. Concurrently, for patients, the platform functions as a support system, to aid them in remembering and managing even complex therapeutic plans.

The online dashboard is developed using the VUE framework, defining a single-page interactive application for physicians. It allows doctors to log in using private credentials and enter prescriptions for the patients. Prescriptions are formulated by the medication name, the corresponding dosage, the designated time of intake, and any relevant instructions (e.g. taken before, during, or after meals). The system allows to define therapies with any combination of schedules during the week, enabling physicians to insert simple daily therapies as well as complex therapies that must be taken at different times during the week. Physicians can also check the adherence of their patients, both global and associated with a specific therapy. Through the online dashboard, data are sent to the cloud database via secure HTTPS communication, ensuring GDPR compliance.

The mobile application is designed as a native application developed using the same framework as the web dashboard, embedded within a VebView environment through the use of the Capacitor tool.^37^ While the mobile app relies on web technologies (HTML, CSS, and JavaScript), it is integrated with capacitor to ensure a native-like experience, allowing patients to install and use the app on Android devices without further requirements. This hybrid architecture reduces development time and effort, as most of the codebase can be reused across platforms. To complement the hybrid approach, Java is used to implement Android-specific functionalities requiring deep integration with the operating system, also including managing background processes, such as handling of precise notifications to ensure timely medication reminders.

**Figure 2.**
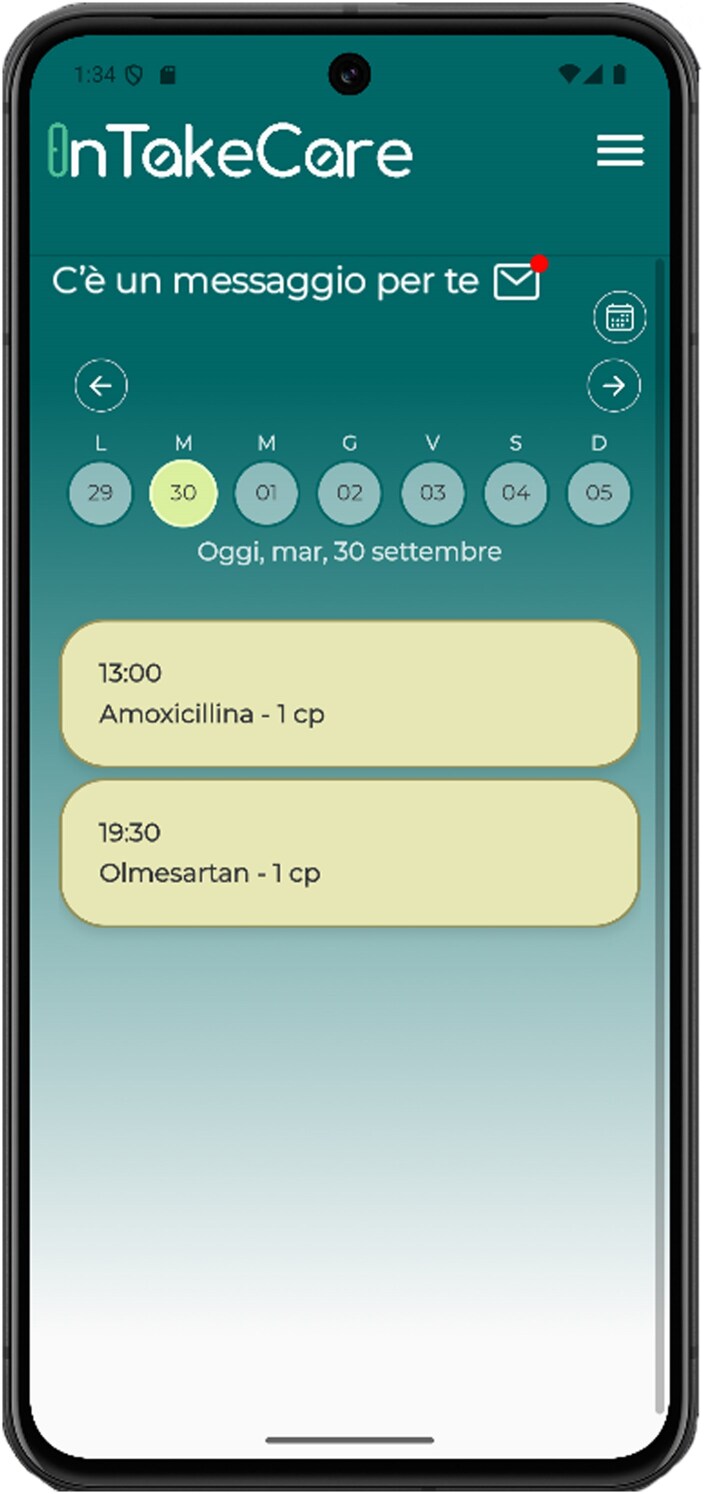
Main page of the InTakeCare mobile application, showing a message notification on top and two medication intakes scheduled for the day.

In *Figure 2*, a screenshot of the InTakeCare mobile application is presented. The home page is in the form of a calendar and offers a detailed overview of the daily medication schedule of the patient. Scheduled intakes for the day are displayed as individual cards with details such as the medication name, posology, scheduled time, and current status (either ‘taken’, ‘missed’, or ‘pending’). Users can navigate through the different days and have a quick access button to return to the current day. Each intake card uses a colour-coded background and icons for increased clarity and ease-of-use. A yellow background indicates that the intake is within the active time window and allows interaction. A green checkmark or a red cross appears when the intake is marked as either taken or missed, respectively.

The card then becomes either completely green or red after the time window is elapsed. The time window for each intake of each therapy is defined by the physician together with the patient, through the web dashboard, with common standard ranges provided for ease of use.

Personalized messages are sent to the patients through the application. Messages are automatically sent by the server according to the frequency identified by the corresponding persona. A visual and auditory notification prompts the user to enter the application and read the message. The list of sent messages is stored in the database to avoid unwanted repetitions. At any point in time, patients are able to access the list of received personalized messages to read them again without limitations.

A ‘diary’ function is also available, enabling patients to add new pages independently. A diary page is composed by

(i) a 5-point Likert scale ranging from ‘very poorly’ to ‘very well’, assessing how the patient feels while completing the diary,
(ii) a self-inserted blood pressure measurement, and
(iii) a slot for freely written notes.

The diary can then be accessed by the physician at a later time through the use of the web dashboard. The purpose of the diary is to enable asynchronous communication with the physician, by providing insight on events that can happen between two cardiologic visits and that would otherwise be easily forgotten.

### Randomized control trial

The clinical validation study is designed as a parallel-group, randomized, open, blinded endpoint, single-centre clinical trial. The study includes adult patients with essential hypertension, in stable clinical conditions, treated with at least one antihypertensive drug for which dedicated plasma essay is available (ramipril, telmisartan, olmesartan, valsartan, amlodipine, atenolol, nebivolol, chlortalidone, hydrochlorothiazide, indapamide, nifedipine, clonidine, canrenone, spironolactone, doxazosin, candesartan, bisoprolol). Only patients with adequate technological tools (Android mobile phone) and skills (ability to use smartphone app) are included. The following exclusion criteria are applied: patients with insufficient technological literacy, dementia or significant psychiatric disorders, conditions significantly limiting vocal communication, pregnancy or breastfeeding, active cancer (except basal cell skin carcinoma), upper limb amputation, and shift workers. All participants must provide written informed consent to be included.

**Figure 3.**
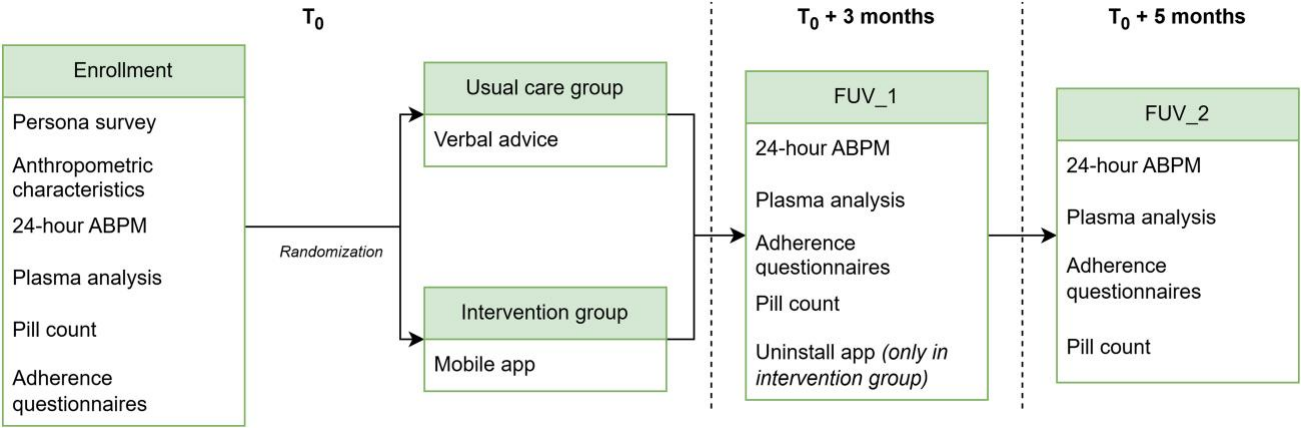
Block schema of the randomized control trial process, highlighting the visits that are performed from start to end. For each visit the actions that are performed are presented, together with the timing of the visit with respect to the starting time for the patient (T_0_).

The participants, consecutively recruited in hypertension clinic, undergo screening and baseline assessment and, if eligible, are randomized to 3 months of active intervention or usual care. A 2-month extension period without intervention (usual care) in either group follows, to assess the persistence of intervention effect over time. In *Figure 3*, a schematic representation of the clinical trial highlighting the actions performed at every step, the timing and the differentiation between usual care and intervention is represented.

The procedures at enrolment include medical history, office blood pressure, anthropometric measures, physical exam, 24-h ambulatory blood pressure monitoring (ABPM) performed with a validated oscillometric device (AND, TM2441, Japan),^38^ venous blood sample for basic biochemical assessments (glycated haemoglobin, creatinine, total, HDL and LDL cholesterol, triglycerides) and for the measurement of plasma levels of antihypertensive medications, pill count (participants are asked to bring all the medications they currently possess and the number of available pills is recorded for each antihypertensive drug). Context information for the interpretation of plasmatic drug levels is also obtained including the timing of last drug intake and food intake.

All participants complete a set of questionnaires composed of both *ad hoc* questions and standardized questionnaires (selected in the previous phases of focus groups and persona construction), based on which the most closely corresponding persona will be identified using the SSML algorithm. Patients are then randomized in a 1:1 ratio to one of the study groups. Randomization is stratified based on their persona, ensuring equality among the number of patients belonging to each persona randomized to intervention and usual care.

In participants randomized to active intervention, physicians create the patient’s account on the web dashboard, inserting all relevant therapies as previously stated. Subsequently, the mobile application is installed and configured on the user’s smartphone, using the created account, so that at the first login, therapies are automatically configured on the user’s smartphone, together with the engagement strategy configuration (messages frequency and content) based on the assigned persona.

The device is set to remind the participant, at the times defined during set-up, which therapy should be taken (drug name and dosage). The participant has 120 min from the first reminder to confirm through the application that the therapy has been taken. After 55 min from the first reminder, a second reminder is sent if the medication has not already been taken. In case the patient took the medication within this window but could not register the intake, he/she may still confirm the intake within the following 10 h, indicating the actual intake time. Otherwise, the intake is considered as missed. All other functions of the application previously described are available to patients at any time.

Participants in the control group receive verbal advice from the physician about the need to follow the treatment recommendations and simple indications on possible solutions to maintain adherence, as by the usual practice of the centre.

First follow-up visit (FUV-1) takes place after 3 months (±2 weeks) with the following assessments: office blood pressure, 24-h ABPM, venous blood sample for plasma levels of antihypertensive medications,^39^ pill count,^40^ and adherence rate registered by the user interface (intervention group only). All participants complete the following questionnaires: MARS5-I,^41^ Hill-Bone Compliance to High Blood Pressure Therapy Scale,^17^ Patient Activation Measurement (PAM) licensed by Insigna Health®,^42^ and in the intervention group participants additionally complete the Twente Engagement with Ehealth Technologies Scale (TWEETS)^43^ and the System Usability Scale (SUS).^44^ The pill count is based on the information collected at baseline, currently available medications, and a log of all additional medication resupplies that took place during the trial period. During the follow-up visit, the app is uninstalled from the devices of participants in the intervention group. Adverse events are collected including serious adverse events and events of special interest.

The final visit scheduled 5 months after randomization (FUV-2) includes office blood pressure, 24-h ABPM, venous blood sample for plasma levels of antihypertensive medications,^39^ questionnaires (MARS5-I,^41^ Hill-Bone Scale,^17^ Patient Activation Measurement (PAM)^42^), and pill count^40^ (see above).

At the end of the study, physicians provide feedback regarding the intervention acceptance on their side.

The efficacy outcomes of the study include (i) primary outcome: percentage of adherent patients based on plasma essay at FUV-1 (after 3 months of randomized intervention; patients are defined as adherent if the drug is detectable in plasma); (b) secondary outcomes—percentage of adherent patients based on plasma essay at FUV-2, percentage of adherent patients assessed with other measures (pill count, self-reported information from user interface, MARS-5I questionnaire), and 24-h blood pressure measured at FUV-1 and at FUV-2. Other outcomes include adherence assessed with Hill-Bone Scale, improvement in patient’s engagement, usability and feasibility of the intervention, and its acceptance by patients in active group and physicians.

The estimated sample size allows to identify a 20% difference in the proportion of adherent participants between usual care and intervention groups, with 80% power and 5% alpha error. Based on these assumptions, 82 participants per treatment arm are required. Considering a 20% dropout rate, the recruitment of 103 participants per group is planned, for a total of 206 treated hypertensive patients.

## Discussion

In this paper, the design of the project aimed to design and clinically validate the InTakeCare platform, a personalized DHS aimed to support and monitor MA in chronic hypertensive patients, is outlined and described. InTakeCare seeks to address the challenges raised by poor MA, leveraging digital health technology, personalization of the intervention, improvement of patient education, and real-time tracking of medication intake.

The DHS that will be developed and evaluated in this study is a beta prototype developed exclusively for use within this research study. It is accessible only to enrolled participants randomized to the intervention arm and is not released for clinical use. At this stage, InTakeCare is not a certified medical device, and no medical claims are made. The purpose of the present study is to evaluate its usability and potential impact on MA to inform a future decision on whether to pursue formal medical device certification.

The study design incorporates principles of user-centred design techniques to engage both patients and healthcare providers in the development process, ensuring that the solution is both practical and scalable in real-world settings.^45^ Furthermore, by implementing persuasive system design techniques, InTakeCare is designed not only to prompt action but also to support positive behavioural changes over time.^46^

Finally, the project design includes both a small-sized pilot phase (during which patients interact with an initial version of InTakeCare and the DHS and personas are refined based on user feedback and observed system usability) and a mid-sized clinical validation study.

The latter follows a randomized, controlled trial design, which remains a gold standard for assessing the efficacy of clinical interventions.

This research is expected to make several key contributions to the fields of MA and digital health. It will generate insights into the behavioural, psychological, and contextual factors that influence medication-taking behaviour. Besides, through the RCT, it will provide empirical evidence on the usability and acceptability of a co-designed mobile intervention for MA, further expanding on current scientific literature about the use of mobile applications within the field by proposing a DHS that implements multiple features previously not present concurrently in other applications, such as personalization of the intervention through the use of personas and real-time physician monitoring through a dedicated dashboard.^47–49^

Beyond the specific use case presented in this study, InTakeCare will provide a transparent, replicable, and theory-informed framework for developing digital health interventions. By documenting the co-design process, the design of the solution and the testing stages, this study offers a practical roadmap for researchers and developers aiming to build patient-centred tools that leverage persuasive system design and behavioural change techniques.^46,50^ This contribution is particularly important as the healthcare sector moves towards more personalized, scalable, and evidence-based DHS.

In conclusion, the implications of this work extend also beyond the context of MA in hypertensive patients. The proposed framework for development can also be applied and extended to other chronic care interventions, potentially improving outcomes in conditions such as diabetes and mental health, opening possibilities for further research. Other studies may explore the long-term clinical impact of the intervention, its cost-effectiveness, integration with healthcare delivery systems including primary care and community health settings, and development of novel modules for patient interaction that further personalize the solution towards the target population.

## Data Availability

No new data were generated or analysed in support of this research.
